# Improved Dimethyl Ether Production from Syngas over Aerogel Sulfated Zirconia and Cu-ZnO(Al) Bifunctional Composite Catalysts

**DOI:** 10.3390/ma16237328

**Published:** 2023-11-24

**Authors:** Hela Lassoued, Noelia Mota, Elena Millán Ordóñez, Sahar Raissi, Mohamed Kadri Younes, Carlos Quilis Romero, Rufino M. Navarro Yerga

**Affiliations:** 1 Laboratory of Material Chemistry and Catalysis, Department of Chemistry, Faculty of Sciences of Tunis, University of Tunis El Manar, Tunis 2092, Tunisia; 2 Instituto de Catálisis y Petroleoquímica (CSIC), c/Marie Curie 2, Cantoblanco, 28049 Madrid, Spaincarlos.quilis@csic.es (C.Q.R.)

**Keywords:** DME, syngas, direct synthesis, Cu-ZnO, sulfated zirconia, sol gel, acidity

## Abstract

This work is dedicated to the study of the effect of the synthesis conditions (drying and calcination) of sulfated zirconia on the final catalytic behavior of bifunctional composite catalysts prepared by the physical mixing of the sulfated zirconia (methanol dehydration catalyst) with Cu/ZnO/Al_2_O_3_ (CZA; methanol synthesis catalyst). The main objective was to optimize the CZA-ZrO_2_/SO_4_^2−^ composite catalyst for its use in the direct production of dimethyl ether (DME) from syngas. Sulfated zirconia aerogel (AZS) and xerogel (XZS) were prepared using the sol–gel method using different solvent evacuation conditions and calcination temperatures, while the Cu-ZnO(Al) catalyst was synthesized using the coprecipitation procedure. The effectivity of CZA-ZrO_2_/SO_4_^2−^ composite catalysts for the direct production of dimethyl ether (DME) from syngas was evaluated in a flow reactor at 250 °C and 30 bar total pressure. The characterization of the sulfated zirconia aerogels and xerogels using different techniques showed that the mesoporous aerogel (AZS_0.5_300) exhibited the best textural and acidic properties due to the gel drying under supercritical conditions and calcination at 300 °C. As a result, the composite catalyst CZA-AZS_0.5_300 exhibited seven times higher DME production than its xerogel-containing counterpart (364 vs. 52 μmol_DME_·min^−1^·g_cat_^−1^). This was attributed to its well-matched metal surface, mesoporous structure, optimal crystallite size and, most importantly, its higher acidity.

## 1. Introduction

Dimethyl ether (DME) is widely recognized as a versatile compound and plays a crucial role in the synthesis of various valuable chemicals, including light olefins, methyl acetate, dimethyl sulfate, acetic acid, oxygenates, as well as serving as a substitute for chlorofluorocarbons (CFCs) in aerosol formulations and a hydrogen feedstock for fuel cell applications [1,2,3,4]. Unlike fossil fuels, DME contains a higher oxygen content of approximately 35% and lacks direct carbon–carbon (C–C) bonds. Instead, it predominantly consists of carbon–hydrogen (C–H) and carbon–oxygen (C–O) bonds, resulting in a lower binding energy. This unique composition gives DME distinct advantages such as reduced ignition delay and a high cetane number (55–60) compared to other fuels [5]. Moreover, DME exhibits the advantage of being non-carcinogenic, low in toxicity, non-corrosive, and easy to store and transport. Additionally, its physical properties bear resemblance to liquefied petroleum gas (LPG), making it an excellent alternative to conventional fuels. Furthermore, the combustion process of DME emits lower levels of hazardous compounds, including CO_2_, as well as primary pollutants such as carbon monoxide (CO), nitrogen oxides (NO_x_), and particulate matter (PM), in comparison to diesel or gasoline [6].

**Scheme 1:**
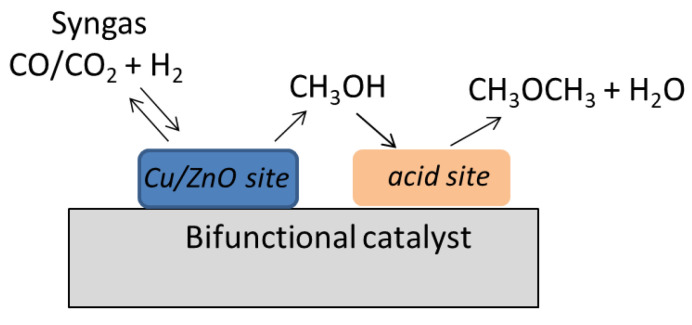
Bifunctional catalysts showing the reaction pathways for the direct production of DME from syngas.

Two distinct pathways are carried out for the synthesis of DME from syngas. The first one, known as the indirect route, involves a two-step industrial process. In this process, methanol is first produced from syngas and purified. Then, it is dehydrated and converted to DME in a separate reactor. The main problem with this two-stage DME process is that it is kinetically limited with a low gas conversion of 15–25% and has a high operating cost [3]. The second alternative method, which is considered more efficient and practical, is the direct route. The direct production of DME in a single reactor using bifunctional catalysts (Scheme 1) reduces the operating cost and it is more thermodynamically favorable [7].

Nowadays, Cu-ZnO(Al) (CZA) catalysts are used for the industrial production of methanol. In these catalysts, metallic Cu constitutes the active sites of the methanol dehydrogenation and ZnO has the remarkable ability to prevent the sintering of copper particles during the calcination process and to improve copper dispersion in the catalyst structure [4,8], while the use of Al_2_O_3_ as a promoter increases the stability and activity of the catalyst [9]. Typically, CZA catalysts are prepared using the co-precipitation method [6], which involves three steps: the precipitation of the precursors to obtain the hydroxycarbonates, the calcination to control the dispersion of CuO-ZnO species, and the reduction of the solid to obtain the active catalyst.

On the other hand, numerous solid acid catalysts have been investigated for methanol dehydration reactions, since it is of interest to researchers focused on the use of alumina and zeolite [10,11]. However, the hydrophilic nature of alumina can pose challenges in terms of water adsorption and the deactivation of Lewis acid sites, affecting catalytic activity, and DME selectivity. Compared to alumina, zeolites with strong Lewis acid sites exhibit superior water tolerance and higher catalytic activity, but their small pores and strong acid sites can also lead to undesirable side reactions and coke deposition, which can adversely affect catalyst stability and performance.

Pure zirconia is chemically unreactive, but after treatment with sulfuric acid, sulfated zirconia becomes a highly reactive super acid catalyst, catalyzing various commercially important reactions such as *n*-alkane isomerization, acylation, alkylation, nitration, etc. [12,13,14,15,16,17,18]. As compared with other acidic solids used for methanol dehydration reactions [12], sulfated zirconia (ZrO_2_/SO_4_) offers several advantages, such as high stability and the flexibility of modification. Its highly acidic or super-acidic character is due to the presence of sulfate anions (S-ZrO_2_), which behave as Lewis and Brønsted sites [12,13]. In fact, sulfated zirconia is a unique acid catalyst that has both types of acid sites due to the electron withdrawing effect of the sulfate groups bonded to the surface of zirconia [14]. In this regard, the work by Witoon et al. demonstrated that catalysts with a low sulfur content (5–10%) mainly presented weak acid sites, acting as Lewis acid sites, while those with a high sulfur content (15–30%) exhibit stronger acidity due to the creation of Brønsted acid sites [15]. Interestingly, it was found that numerous mesopores were created on the surface of the catalyst when a small amount of sulfur was added. Several preparation methods have been used for the synthesis of sulfated zirconia, such as sol–gel [19,20,21], precipitation [22], or impregnation [23] methods, and each of these methods leads to different physicochemical properties of the acidic solid, and in consequence, to different catalyst activities. In particular, the structural and textural properties of the sulfated zirconia were found to be affected by the synthesis strategies used [20]. As compared to the precipitation of an aqueous zirconium salt, the more common sol–gel technique is advantageous because it allows for better control of the synthesis process, leading to better physical characteristics of the final acid solid [20]. In the one-step sol–gel method, the hydrolysis of the metal alkoxide and sulfation with sulfuric acid is performed simultaneously. In the two-step sol–gel preparation, firstly, the formation of sol occurs via the hydrolysis of the metal alkoxide. After this first step of condensation, the formation of a three-dimensional network gel of zirconium hydroxide occurs [20]. The effect of different metal sulfate precursors on the structural and catalytic performance of zirconia in the dehydration of methanol to dimethyl ether was studied by Said et al. [21]. The best catalytic activity result obtained for the sulfated zirconia prepared using a CuSO_4_·5H_2_O precursor was explained in the following way: the presence of Cu acts as a site for oxygen removal from the bulk zirconia to create additional Lewis acid sites [21].

The use of sulfated zirconia for designing novel bifunctional catalysts for the direct synthesis of DME from syngas is promising and worth exploring, but its synthesis should be optimized. Since no study has been published on the effect of solvent evaporation on its catalytic activity, this prompted us to prepare the sulfated zirconia using the sol–gel method using two different solvent evaporation procedures and calcination temperatures to optimize its acidic and structural properties. The synthetized sulfated zirconia aerogel and xerogels were then physically mixed with a Cu-Zn(Al) methanol synthesis catalyst to obtain an effective catalyst for the production of DME from syngas. The catalytic activity results and the characterization of the catalysts using various techniques clearly demonstrated the superiority of the CZA catalysts mixed with a sulfated zirconia mesoporous aerogel over its mesoporous/microporous xerogel counterpart.

## 2. Materials and Methods

### 2.1. Catalyst Preparation

#### 2.1.1. Preparation of Sulfated Zirconia (ZrO_2_/SO_4_^2^) Acid Catalysts

Aerogel and xerogel sulfated zirconia (ZrO_2_/SO_4_^2^) solids were prepared following the sol–gel method, as previously described [24]. To begin, the zirconium propoxide (70% in propanol, (Sigma-Aldrich, Milwaukee, WI, USA) was dissolved in 1-propanol (ACROS 99%, Across Organics, Antwerp, Belgium). Then, concentrated sulfuric acid was added with a molar ratio $\frac{n_{S}}{nZr}=0.5$ and was stirred well for 1 h. At last, the distilled water was added to the solution with a hydrolysis ratio of $\frac{n_{H2O}}{nZr}=$3. Then, the mixture was kept under agitation at room temperature until the obtaining of the gel. The resulting gel was subdivided into two parts: the first one was dried in an oven overnight under atmospheric pressure at 110 °C to obtain the xerogel sample, and the second part was dried under supercritical conditions of the solvent in an autoclave (263.3 °C, 51 bar) to obtain the aerogel. Then, both catalysts were calcined at 300 °C or 560 °C to eliminate the residues of the carbonaceous compounds. Hereafter, the aerogel and xerogel acid catalysts will be denoted as AZS0.5T and XZS0.5T, where A denotes aerogel, X denotes the xerogel, Z denotes zirconia, S0.5 is the sulfate–molar ratio, and T is the temperature of calcination.

#### 2.1.2. Preparation of Cu-ZnO-Al (CZA) Catalyst

The Cu-ZnO(Al) catalyst, referred to as CZA, was prepared using an optimized co-precipitation method [25] using Cu, Zn, and Al aqueous nitrate solutions (1 M) with the composition (Cu/Zn/Al = 68/29/3) under a controlled pH = 6.5, and temperature (65 °C), as proposed previously. All the precursors were purchased from Sigma-Aldrich, Milwaukee, WI, USA as follows: Cu(NO_3_)_2·_7H_2_O, 99.999%, Zn(NO_3_)_2_·6H_2_O, 99.999%, Al(NO_3_)_2_·9H_2_O (99.997%), and Na_2_CO (99.999%). The obtained precipitates were aged, filtered, and washed with deionized water, and then dried at atmospheric pressure at 80 °C for 12 h and calcined under air at 340 °C for 2 h with a heating rate of 2 °C/min.

#### 2.1.3. Preparation of CZA-ZrO_2_/SO_4_ Bifunctional Composite Catalysts 

The CZA-ZrO_2_/SO_4_ bifunctional composite catalysts were prepared by the physical mixing of aerogel or xerogel sulfated zirconia (calcined at T = 300 °C) with the CZA solid in a mass ratio of 1:2. It is well known that the characteristics of bifunctional catalysts are highly dependent on the mixing process (manual grinding with mortar, agitation, ultrasound, or balls) and the applied forces [26,27]. Thus, to avoid changes of the catalyst surface properties, both of the solids were mixed in agate mortar for a short time (5 min) and without hard pressing to form the homogenous mixture.

#### 2.1.4. Catalysts Characterization

The surface area and average pore diameter of the catalysts were determined from N_2_ adsorption–desorption data using a Micrometrics ASAP 2100 automatic device (Micromeritics, Norcross, GA, USA). The Brunauer–Emmett–Teller (BET) and the Barrett–Joyner–Halenda (BJH) methods were applied to evaluate the surface area and the pore size distribution, respectively [28].

The influence of the calcination temperature on the stability of the SO_4_^2−^ groups of the aerogel and xerogel sulfated zirconia was determined using a thermogravimetric (TGA/DTA) analysis carried out on a Model TGA 2950 high-resolution thermogravimetric analyzer (TA Instruments, New Castle, DE, USA) by heating the samples under an He flow from 30 to 900 °C at the heating rate of 5 °C·min^−1^.

The X-ray diffraction (XRD) data were recorded using an X’Pert Pro-Panalytical diffractometer (Malvern Panalytical Ltd; Malvern, UK) with Ni-filtered Cu Kα radiation (λ = 1.5418 Å). Measurements were taken between 5 and 90° (2θ). The crystallite size of the samples was calculated using the Debye–Scherrer Equation (1):

(1) $$D=\frac{K\lambda }{\beta cos\theta }$$

where *D* is the crystallite size (nm), K represents shape factor (0.9), λ is the wavelength of the X-ray of the applied source, β is the full width at half maximum of the most intense peak (FWHM) in radian, and θ is the Bragg diffraction angle in radian. As well, the crystallite size of CuO was estimated from the full width at half maximum (FWHM) of the CuO peak characterized by diffraction plane (111) by using the Debye–Scherrer equation with a correction for the instrumental broadening.

The ATR-FTIR spectra of the samples were analyzed using a JASCO FT/IR-6300 Fourier-transform (FT) IR spectrometer (JASCO corp. Tokyo, Japan) and were recorded in a scan range from 4000 to 400 cm^−1^. For the Attenuated Total Reflection (ATR) mode FTIR measurements, the acid samples were diluted with potassium bromide. For the acid samples, we used 1 mg of acid catalyst and 19 mg of KBr, while for the bifunctional catalysts we used 4.8 mg of catalyst (mixture of 33.33% and 67.67% of acid and methanol catalysts, respectively).

The reducibility of the pure CZA and combined catalysts was measured using a temperature-programmed reduction (TPR) analysis carried out in a semiautomatic PID Eng. Tech (Madrid, Spain) apparatus equipped with a U-shaped quartz reactor and a thermal conductivity detector. The samples were initially flushed under an He flow (30 mL/min) at 120 °C for 20 min in order to remove water and clean the surface. Subsequently, the temperature was increased from room temperature to 200 °C at a ramp rate of 2 °C/min under a 10%H_2_/Ar flow (50 NmL/min) to obtain the TPR profiles of the reduced samples.

The Cu surface area (S_Cu_) and Cu crystallite size were estimated from the N_2_O decomposition reaction. After reduction, samples were treated under an N_2_O/Ar flow (2 vol%, 30 mL/min) at room temperature for 5 s. After chemisorption, the He flow (30 mL/min) was passed for 30 min to weed out the physisorbed N_2_O. Subsequently, the sample was reduced under an H_2_/Ar flow (10 vol.% H_2_, 50 mL/min) at a rate of 10 °C/min until 200 °C. A molar stoichiometry ratio of Cu(s)/N_2_O = 2 was assumed to calculate the copper surface area in the combined catalysts, where Cu(s) referred to the superficial copper atom. An average value of 1.46 × 1019 copper atoms/m^2^ was used for the surface density of the copper metal.

The acidity of the acid and mixed catalysts was determined using a temperature-programmed desorption of ammonia (NH_3_-TPD), which was recorded on a Micromeritics TPD/TPR apparatus (GA, USA) with a mass spectrometer (MS). All the samples (100 mg) were pretreated under an He flow (30 mL/min) at 160 °C for 20 min, and then, the temperature was decreased to 100 °C under the He flow. After that, a flow of ammonia (5 vol.%) was introduced at 100 °C. After the saturation of the sample, the physisorbed NH_3_ was removed with an He flow (30 mL/min) at 100 °C for 40 min, and thereafter, the desorption of ammonia was performed by heating the sample from 100 to 800 °C at a rate of 10 °C/min.

### 2.2. Catalytic Activity in the Direct Synthesis of DME from Syngas

The performance of the bifunctional composite catalysts of CZA and sulfated zirconia for the synthesis of DME from syngas was studied in a fixed-bed reactor loaded with 0.225 g of catalyst (grain size of 0.30–040 mm) diluted with silicon carbide SiC (1:3 vol, the same grain size as catalyst) to withstand temperature gradients. The reduction of the catalysts was performed in situ under a hydrogen flow (2.2% H_2_/Ar, 50 mL/min) based on two temperature steps: 150 °C (2 °C/min) and subsequent heating at 200 °C (1 °C/min) for 2 h. After the reduction, the reactor was fed with a mixture of the syngas (75 NmL/min, molar composition: 4.5% CO_2_, 22.0% CO, 58.8% H_2,_ and 14.7% N_2_). Activity was measured at 250 °C and 30 bar for 3 h after stabilizing the catalyst for 1 h under syngas feed. A GC (Varian 450-GC (Varian Inc. CA, USA) with a TCD equipped with capillary columns connected in series (QS-bond: CO_2_, CH_3_OH, DME and H_2_O and molecular sieve 5A: H_2_, O_2_, N_2_, CO) was used to analyze the products. The CO conversion, CO_2_ DME, CH_3_OH selectivity, and production rate (space-time yield, STY) were calculated according to Equations (2)–(7):

CO conversion:

(2) $$X_{CO}=\frac{MCO\left(i\right)-MCO(f)}{MCOx(i)}\cdot 100$$

CO_2_ selectivity:

(3) $$S_{{CO}_{2}}=\frac{M_{{CO}_{2}}}{\sum Mproducts}\cdot 100$$

DME selectivity:

(4) $$S_{DME}=\frac{M_{DME}}{\sum Mproducts}\cdot 100$$

CH_3_OH selectivity:

(5) $$S_{{\mathrm{CH}}_{3}OH}=\frac{M_{{\mathrm{CH}}_{3}OH}}{\sum Mproducts}\cdot 100$$

DME production rate:

(6) $${STY}_{DME}=\frac{MDME(mol)}{mcat\left(g\right)\cdot t(min)}$$

CH_3_OH production rate:
  (7) $${STY}_{{\mathrm{CH}}_{3}OH}=\frac{{\mathrm{MCH}}_{3}OH(mol)}{mcat\left(g\right)\cdot t(min)}$$
  where M refers to the molar flow rate of each product.

## 3. Results

### 3.1. Characterization of Sulfated Zirconia Acid Catalysts 

#### 3.1.1. Textural Properties

**Figure 1:**
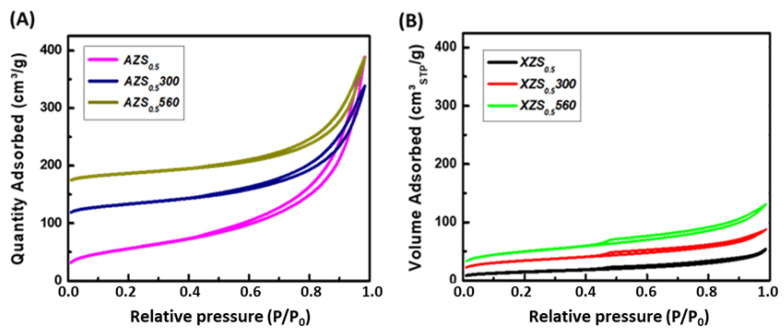
N_2_ adsorption–desorption isotherms of sulfated zirconia aerogels (**A**) and xerogels (**B**) dried and calcined at different conditions.

**Figure 2:**
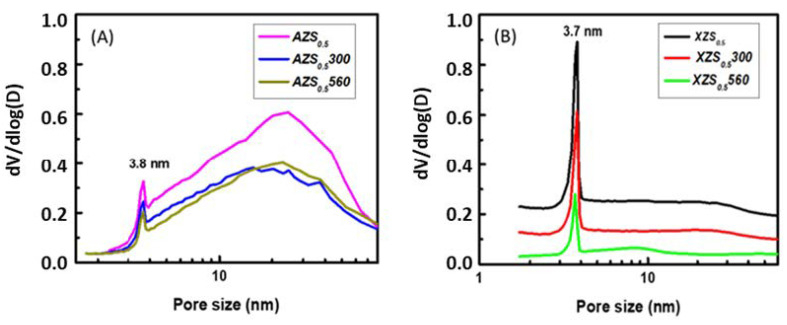
Influence of calcination temperature (300 vs 560 °C) on the BJH desorption pore-size distribution of sulfated zirconia aerogels (**A**) and xerogels (**B**).

The acid sulfated zirconia (ZrO_2_/SiO_4_^2−^) was prepared using the sol–gel method followed by thermal drying or calcination. The effect of the thermal drying for the evaporation of the solvent on the textural properties of the aerogel and xerogel of the sulfated zirconia was studied using N_2_ adsorption–desorption isotherms at −196 °C. The N_2_ adsorption–desorption isotherms and the pore size distribution of the synthesized aerogel and xerogel materials before and after their calcination at different temperatures are presented in Figure 1A and Figure 1B, respectively. Based on the IUPAC classification, the adsorption branch of the dried aerogel (AZS_0.5_) is a type IV(a) isotherm with a type H3 hysteresis loop, which is typical for mesoporous materials formed by non-rigid aggregates of plate-like particles or macropores that are partially filled with pore condensate [28]. The sharp increase at a high relative pressure may indicate the presence of slit-like pores. In contrast to aerogel sulfated zirconia, the N_2_ adsorption isotherm of the dried xerogel sulfated zirconia is of type I with a plateau at higher relative pressures and no hysteresis loop (Figure 1B), which is typical of microporous materials without mesoporosity [28]. The adsorption branch of both the aerogel (AZS) and xerogel sulfated zirconia (XZS) samples, following calcination at 300 °C, conforms to the typical Type IV(a) isotherms. The H3 hysteresis loop exhibited by the aerogel is characteristic of mesoporous materials formed by loosely associated plate-like particles or macropores that contain some pore condensate. The notable upswing at higher relative pressures suggests the existence of slit-like pores. These findings imply a relatively uniform pore size and shape within the material, facilitating interconnectivity for capillary condensation within the mesopores. In contrast, the hysteresis loop of the xerogel solid conforms to Type H4, implying the formation of a material containing a mixture of micro, meso, or possibly macroporosity. The Type H4 hysteresis loop is typically observed in materials with a wider spectrum of pore sizes and configurations. This is a consequence of the intricate structure of the material, which comprises interlinked pores of varying dimensions and geometries. N_2_ adsorption–desorption isotherms play a key role in the analysis of the structural characteristics of porous materials. In this context, it reveals that the aerogel predominantly exhibits mesoporosity, featuring non-rigid assemblies of plate-like particles. Conversely, the xerogel has a more intricate pore structure, encompassing both micro and mesopores, and even possibly macropores. The heterogeneity of the xerogel surface is confirmed by analyzing the BJH desorption pore-size distribution data, as shown in Figure 2B. It reveals a pronounced Kelvin peak at approximately 4 nm. This phantom peak arises when certain exceedingly narrow pores are incompletely emptied during desorption due to the influence of capillary forces and gas adsorption within its very tiny pores. These pores are so slender that, even as the pressure decreases, a fraction of the gas remains confined within them. This disparity can be primarily attributed to the inevitable phenomenon of pore shrinkage associated with xerogels, stemming from capillary forces, solvent evaporation, and surface tension forces. During solvent evaporation, an interface between the solvent and air forms within the pores, resulting in a surface tension that exerts significant capillary forces, causing the contraction of pore walls. This results in the compression of the porous structure, culminating in a reduction in pore volume and an overall densification of the xerogel material [24]. For both the aerogel and xerogel, an increase in the calcination temperature from 300 °C to 560 °C led to a decrease in the specific BET surface area and total pore volume. Taking into account the study of Mohammadi et al. [29], the observed decrease in the specific BET surface area could suggest an increase in the porosity of the samples caused by the ihydroxylation process.

Regardless the sample calcination temperature, the aerogel and xerogel sulfated zirconia exhibit very different pore-size distributions, which results from applying the BJH model to the desorption branch of the N_2_ isotherm (Figure 2A and Figure 2B, respectively). The heterogeneity of the xerogel surface is confirmed by analyzing the BJH desorption pore-size distribution data, as shown in Figure 2B. As can be seen, unlike the xerogel, the aerogel shows a very broad pore-size distribution coming from the interparticle voids [28]. As a consequence, all aerogels exhibit a much larger average pore size than xerogels (11.0–13.8 nm vs. 4.7–6.4 nm).

In this work, the BET model and t-plot method were used for comparative purposes and the data obtained are recompiled in Table 1. As can be seen, regardless of the calcination temperature, all aerogel sulfated zirconia samples exhibit a higher specific surface (S_BET_), total pore volume (V_total_), and average pore diameter (d). As compared with the dried samples (AZS_0.5_ and XZS_0.5_), all calcined materials exhibit a much lower S_BET_ due to some textural evolution during calcination. This is because the removal of the solvent in an oven at an atmospheric pressure via a simple evaporation process creates a liquid–vapor interface inside the gel, which generates a surface tension that influences the pores and leads them to decrease. However, removing the solvent under supercritical conditions avoids the phenomenon of surface tension, which could lead to the non-shrinking of the pores [14,24]. Since both AZS_0.5_ and XZS_0.5_ calcined at 300 °C have similar specific BET surface areas (126 vs 116 m^2^·g^−1^) and different porosity characteristics, these two materials were selected for the preparation of CZA-ZrO_2_/SO_4_ composite bifunctional catalysts.

#### 3.1.2. TGA/DTA 

**Figure 3:**
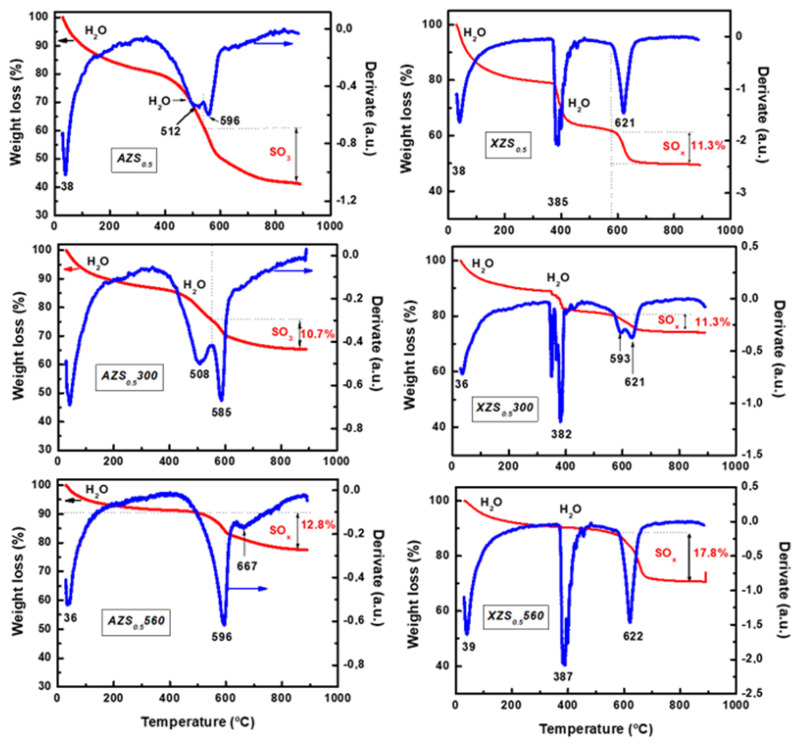
The TGA and DTA curves for dried and calcined aerogel (AZS) and xerogel (XZS) samples.

The stability of the SO_4_^2−^ groups of aerogel and xerogel sulfated zirconia was evaluated via a thermogravimetric analysis (TGA/DTA) by heating the samples in helium from room temperature up to 900 °C. Figure 3 compares the TGA and DTA profiles of the dried and calcined samples. The TGA curves of the dried samples indicate a high total weight loss (<50%), which starts immediately with increasing temperature. Compared to the dried samples, the calcined samples show a lower weight loss due to the previous loss of water during calcination. In general, the aerogel shows a higher weight loss than the xerogel, suggesting its lower hydrophobicity. For both sulfated zirconia aerogels and xerogels, weight loss before 350 °C is associated with the evaporation of water adsorbed on the solid surface [30], whereas the weight loss observed in the xerogel samples between 350 and 400 °C is due to the removal of water from the crystal lattice and the desorption of hydroxyls [30]. Finally, the third weight loss above 400 °C is due to the evolution of sulfur oxides (SO_x_) from the thermal decomposition of the surface sulfated groups [30,31]. Because sulfur species that contain more oxygen decompose more easily [30], the peaks around 500 and 580–620 °C could be attributed to the evolution of SO_3_ and SO_2_, respectively. The percentage of total weight losses (H_2_O + SO_x_) and the evolution of the SO_x_ species are given in Table 1. As can be seen, the xerogel shows a higher evolution of sulfur oxides than the aerogel. Because the loss of sulfur allows the exothermic transition of the tetragonal phase to the monoclinic phase of zirconia [24], this transformation could be easier for the xerogel than for the sulfated zirconia of the aerogel.

#### 3.1.3. XRD Characterization

**Figure 4:**
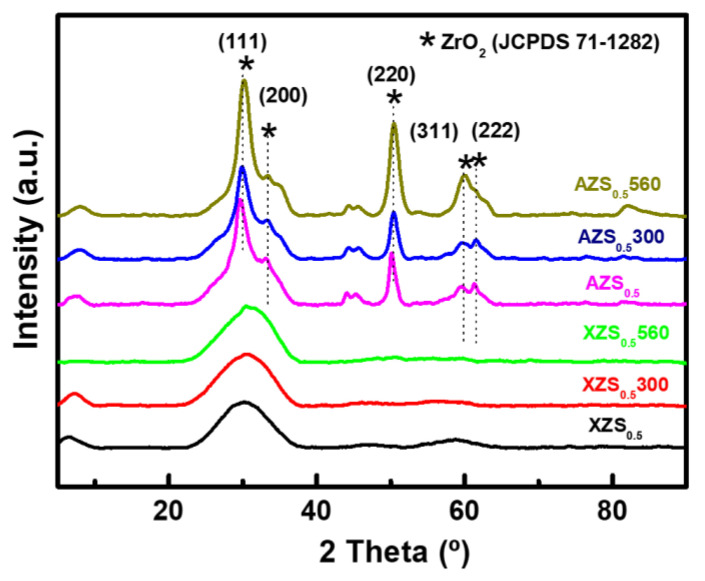
XRD patterns of aerogel (AZS) and xerogel (XZS) sulfated zirconia before and after calcination at 300 and 560 °C.

Figure 4 shows the X-ray diffraction patterns of dried and calcined aerogel and xerogel sulfated zirconia solids. The xerogel samples obtained by solvent drying in an oven present widened and broad peaks between 20 and 40° and 40 and 65°, revealing an amorphous structure with a poorly developed tetragonal ZrO_2_ phase [32]. This is expected because the sulfated zirconia started to form crystals after calcination at 500 °C and its crystallization increases with an increase of the calcination temperature [33]. Contrary to the xerogel sulfated zirconia, the XRD pattern of the aerogel samples obtained by solid state drying under supercritical solvent conditions showed intense and sharp peaks at 2θ = 29.65, 33.18, 50.07, 59.28, and 61.27°, corresponding to the tetragonal phase of zirconia assigned to the (111), (200), (220), (311), and (222) planes, respectively, with a space group of P42/nmc (JCPDS card no. 71-1282) [34], and a poorly developed monoclinic phase, with a space group of P21/C (JCPDS card no. 83-0943). The sulfate ions on the surface of the zirconia induces the structural stabilization of the tetragonal phase and suppresses the crystallite growth [35,36]. This stabilization is observed in the X-ray diffraction results, as shown in Figure 4 in the case of the aerogel catalysts, which maintain the tetragonal phase even after the drying method and the calcination step, while the xerogel catalyst showed the poor crystalline development of this phase.

The adsorption of oxygen at the oxygen-deficient surface sites of zirconia could be the main reason for the transition from the tetragonal to the monoclinic phase. However, when sulfate ions are present on the catalyst surface, they shield the surface from oxygen adsorption and prevent this transition [37]. In addition, the tetragonal phase is thermodynamically stable when the crystallite size becomes smaller than 30 nm [38], which is the case in the aerogel catalyst with a crystallite size of 6 nm, which was calculated using the Debye–Scherrer equation. The tetragonal crystalline form of zirconia is known to be associated with improved acidity [39]. Tetragonal zirconia crystallites would have a lower surface/interface energy than monoclinic crystallites [40]. As well, the acidic character of sulfated zirconia is also related to the predominance of OH groups attached to the Zr atom (type II) of the tetragonal zirconia and the nature of the hydroxyl group on the surface of sulfated zirconia [41].

#### 3.1.4. FTIR Study

**Figure 5:**
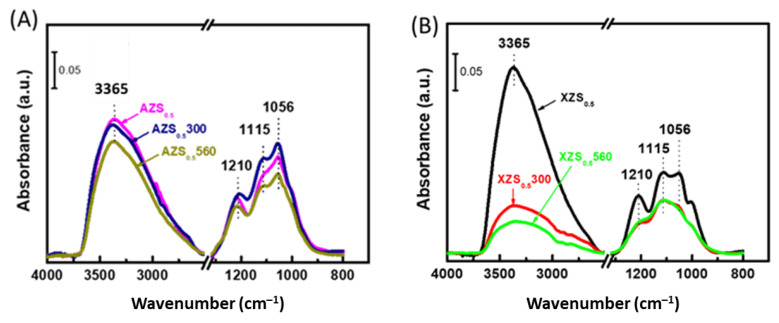
ATR-FTIR spectra of sulfated zirconia aerogel (**A**) and xerogel (**B**) before and after calcination at 300 and 560 °C.

Figure 5A,B display the FTIR spectra of the dried and calcined aerogel and xerogel sulfated zirconia. All the spectra showed broad bands with the maximum centered at 3365 cm^−1^, which is attributed to the vibration bands of the hydroxyl group of the physisorbed water and hydroxides [42,43]. There are three types of surface hydroxyl groups on the surface of ZrO_2_: terminal, tri-bridged, and surface-bound hydroxyl groups [44]. In the case of both acid catalysts, the peaks appear in the 3200–3600 cm^−1^ region, indicating that the water molecules are simply bounded on the ZrO_2_ surface, forming surface-bound hydroxyl groups (OH_w_). It is worth noting that the bands associated with physisorbed water are more pronounced in the case of the xerogel dried at 110 °C, which could be explained by its non-treated surface on which the water is easily adsorbed. In addition, all the spectra showed characteristic bands of inorganic chelating bidentate sulfate ions coordinated to zirconium cation, situated at 900–1245 cm^−1^ [45,46]. For the xerogel samples, the intensity of these bands reduced upon calcination due the decreased concentration of the sulfate species due to its decomposition. Upon calcination at 300 °C, a broad band associated with the characteristic sulfate species appears instead of clear bands. As for the aerogel samples, the characteristic bands of the sulfate species are still clear even after calcination at 560 °C. These results indicate the enhanced stability of the sulfate species in the case of the aerogel, suggesting a strong interaction between the sulfate species and zirconia [31]. The bands of oxygen associated with zirconium (Zr-O-Zr) are situated at 648 cm^−1^ and 639 cm^−1^ and are not shown here.

#### 3.1.5. TPD/MS of NH_3_

**Figure 6:**
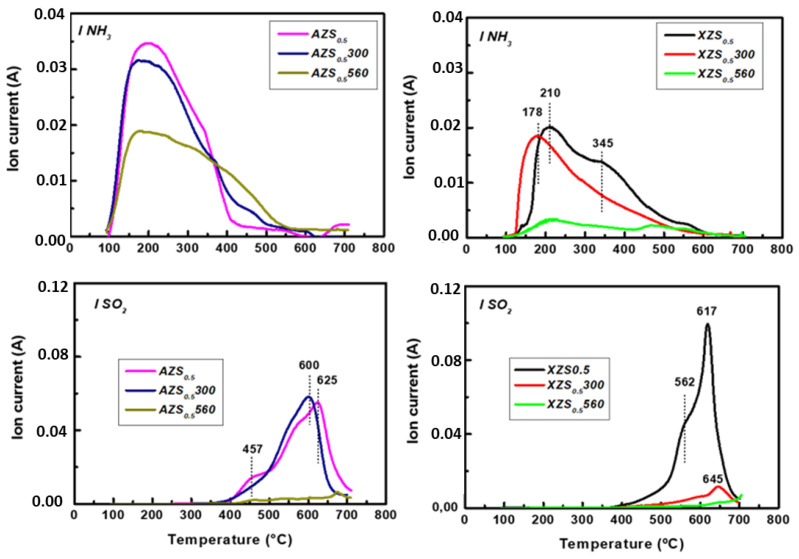
NH_3_-TPD/MS profiles (m/e = 17) and SO_2_ signals (m/e = 64) recorded during NH_3_-TPD/MS of sulfated zirconia aerogels and xerogels dried and calcined at 300 and 560 °C.

The strength of the acid sites and the nature of the desorbing species after the drying and calcination of the aerogels and xerogels were studied using a temperature-programmed desorption of ammonia (NH_3_-TPD) followed by a mass spectrometry. NH_3_-TPD/MS profiles of aerogel and xerogel sulfated zirconia are shown in Figure 6, respectively. Besides the desorbed NH_3_ (m/e = 17), the TPD/MS analysis revealed the evolution of SO_2_ (m/e = 64), CO (m/e = 28), and CO_2_ (m/e = 44). The presence of the ion signal of CO (m/e = 28) and CO_2_ (m/e = 44) is due to the oxidation of the residual organic species, which are still present on the catalyst surface after calcination at 300 and 560 °C.

The NH_3_-TPD/MS profiles of the aerogels and xerogels are very different, indicating their different acidity. Based on the ammonia temperature desorption, the acid sites of the sulfated zirconia samples could be classified as weak acid sites (T< 200 °C), medium acid sites (200 < T < 400 °C), and strong acid sites (> 400 °C) [47,48]. The low temperature peak of the ammonia TPD is only due to the ammonia species adsorbed on the weak acid site. However, the peak corresponding to the high temperature is controlled by both the acid strength and the amount of acid due to ammonia readsorption [49]. Regardless of thermal treatment, the aerogels exhibit a higher acidity than the corresponding xerogels (Table 2). For example, the crystalline aerogel calcined at 300 °C exhibited a higher amount of weak and medium strength acid sites than the amorphous xerogel calcined at the same temperature. This result contrasts with the data from Rachmat et al., who observed that amorphous sulfated zirconia calcined at 400 °C adsorbs more ammonia than other more crystalline catalysts; this result is explained by the presence of hydroxyl groups bonded to amorphous Zr which are incompletely eliminated during calcination at 400 °C [33]. Since hydroxyl groups are suitable for the chemisorption of ammonia [33], we deduce that crystalline aerogel might have a higher amount of hydroxyl groups than amorphous xerogel. In addition, unlike XZS_0.5_300 xerogel, crystalline AZS_0.5_300 aerogel has an important amount of medium acid sites.

It is well established that sulfated-ZrO_2_ solids contain both Brønsted (H^+^) and Lewis (Zr^+^) acid sites [11,50]. The concentration of these sites can be influenced by the presence of sulfate ions (SO_4_^2−^) on the surface of the catalyst. This is because the sulfate species (SO_4_^2−^) have the ability to create Lewis acid centers on the surface of the ZrO_2_ catalyst through the bidentate covalent character of SO_4_^2−^ coordinated with Zr^4+^ ions via the strong electron-withdrawing nature of the sulfate group, which plays a significant role in the generation of highly acidic active sites on sulfated zirconia catalysts [51]. Thus, an optimal concentration of sulfate ions leads to an increase in the number of active acid sites, including both Brønsted and Lewis acid sites. It is well known that at a high temperature, the decomposition of sulfated zirconia leads to the evolution of SO_2_ species [52]. The calcination at a high temperature causes the dehydration of Brønsted acid sites, leading to the loss of protonic sites and loss of (SO_4_^2−^) groups on the surface of the catalyst (Figure 3). As can be seen, the increase in the temperature of calcination led to a decrease in the overall catalyst activity of the composite catalysts. In good agreement with the study by Rachmat et al. [33], an increase in the calcination temperature from 300 to 560 °C led to a large decrease in the amount of adsorbed ammonia. This is probably because the decrease in the specific surface area of the aerogel (Table 1) caused a decrease in ammonia adsorption. In addition, this is because the calcination led to the repelling of the sulfate groups from the pores to the surface of the zirconium, which was previously observed by Tyagi et al. [53].

Considering the intensity of the SO_2_ evolution peaks during the NH_3_-TPD experiments (Figure 6), the aerogels show a higher SO_2_ evolution than their xerogel counterparts. The SO_2_ evolution profiles in Figure 6 proves that the aerogel sample possessed a more important amount of SO_4_^2−^ species, which decomposed at a lower temperature than in the case of the xerogels. The ion signal of the sulfate species of the aerogel and xerogel catalysts presents a shoulder in the low-temperature region, suggesting that the thermal stability of SO_2_ is not homogeneous [54]. These results prove that the mode of solvent removal during the preparation step of the sulfated zirconia samples affects both the acidity and the stability of the SO_4_^2−^ ligand of the acid solids. The higher concentration of sulfate ions on the surface of the zirconia was observed in the cases of the uncalcined xerogel and in the aerogel sample calcined at 300 °C.

### 3.2. Characterization of CZA-ZrO_2_/SO_4_ Bifunctional Composite Catalysts

#### 3.2.1. XRD Characterization of Bifunctional Calcined Catalysts 

**Figure 7:**
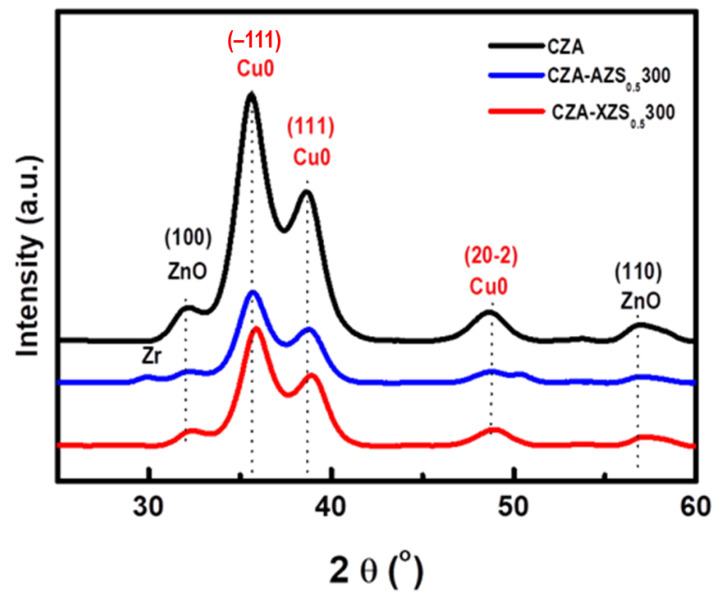
XRD patterns of calcined aerogel (CZA-AZS_0.5_300) and xerogel (CZA-XZS_0.5_300) composite catalysts. CZA catalyst is used as reference.

As mentioned above, the sulfated zirconia samples AZS_0.5_ and XZS_0.5_ calcined at 300 °C were selected for the preparation of CZA-ZrO_2_/SO_4_ bifunctional composite catalysts because they have similar specific BET surface areas (126 vs 116 m^2^·g^−1^) and different porosity characteristics. The crystal structures of the bifunctional composite catalysts prepared with physical mixing were investigated using powder X-ray diffraction. Figure 7 shows the XRD diffractograms of the CZA-AZS_0.5_300 and CZA-XZS_0.5_300 composite catalysts and the CZA catalyst for the methanol synthesis as a reference. The XRD profile of the calcined CZA catalyst presents the typical peaks characteristic of the monoclinic CuO phase (space group: C2/c; JCPDS 01-080-0076) at 2θ = 35.54° (−111), 38.64° (111), and 48.85° (−202) and the peaks characteristic of the ZnO phase (JCPDS 36-1451) at 2θ = 31.96°, 34.38°, 36.24°, and 56.95°. However, the peaks of ZnO at 2θ = 34.38° and 36.24° cannot be discerned due to the overlapped signal of CuO. The broad diffraction suggests that a portion of the copper might be dissolved in the Zn matrix or that the CuO phase is in close contact with the ZnO phase [55]. No diffraction peaks related to Al_2_O_3_ could be observed, due to its presence in an amorphous state, or since it might be highly dispersed in the catalyst [56]. The crystallite size of CuO and ZnO in the CZA catalysts calculated using the Debye–Sherrer equation is around 5.4 and 6.0 m, respectively. However, when CZA was mixed with acid catalysts, the intensity of the CuO and ZnO diffraction peaks decreased with respect to the CZA reference as a result of the dilution with the acid catalyst [57,58]. No significant changes in the diffraction profiles of the CuO and ZnO phases are observed after contact with the acid catalysts, indicating the absence of strong interactions between the methanol synthesis and the acid functionalities. As for ZrO_2_ tetragonal phase, only the pattern of CZA mixed with the aerogel acid catalyst CZA-AZS_0.5_300 presented a very low intensity peak at 2θ = 30°.

The crystalline domains of CuO and ZnO in the bifunctional catalysts are similar to those observed in the individual CZA reference (Table 4). The crystallite size of CuO determined from the 100 plane using the Debye–Scherrer equation shows the same crystallite size, around 4 nm in the bifunctional catalyst. No significant changes were noticed in the crystallite size of ZnO after hybridization.

#### 3.2.2. FTIR of Bifunctional Calcined Catalysts 

**Figure 8:**
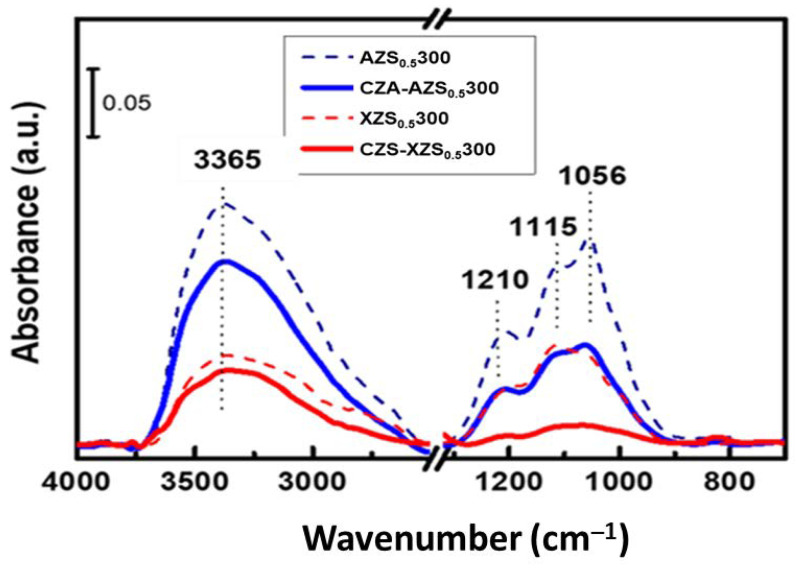
ATR-FTIR spectra of aerogel and xerogel sulfated zirconia (calcined at 300 °C) before and after their physical mixing with metallic CZA methanol catalyst to obtain bifunctional composite catalysts.

The changes in the hydroxyl region of the sulfated zirconia after physical mixing with the CZA catalyst were studied using an ATR-FTIR spectroscopy. Figure 8 compares the FTIR spectra of aerogel and xerogel sulfated zirconia calcined at 300 °C with those of corresponding composite catalysts. The peaks appearing in the hydroxyl region (3200–3600 cm^−1^ region) of both composite catalysts and their corresponding acid solids are due to the water molecules bounded on the ZrO_2_ surface forming surface-bound hydroxyl groups (OH_w_). The broad bands with the maximum centered at 3369 cm^−1^ and at 3270 cm^−1^ are attributed to the vibration bands of the hydroxyl group of the physisorbed water and hydroxides [42,43], which are accompanied with one band situated at 1636 cm^−1^ (not shown here) assigned to the bending mode of adsorbed water. It should be noted that the mesoporous composite CZA-aerogel catalyst exhibits a much higher amount of surface hydroxyl groups than its xerogel counterpart. In addition, the bands of inorganic chelating bidentate sulfate ions coordinated to zirconium cation are observed in the 900–1245 cm^−1^ region [44]. As expected, after the physical mixing of CZA with acidic aerogel and xerogel sulfated zirconia, the intensities of all bands decreased very much due to the dilution effect. The CZA-AZS_0.5_300 and CZA-XZS_0.5_300 bifunctional catalysts prepared with simple mixing show no noticeable change in the surface sulfate ions of the sulfated zirconia; therefore, the acidity associated with the surface sulfate ions was not significantly modified in the bifunctional catalysts compared to the bare acidic sulfated zirconia.

#### 3.2.3. TPD/MS of Adsorbed NH_3_

**Figure 9:**
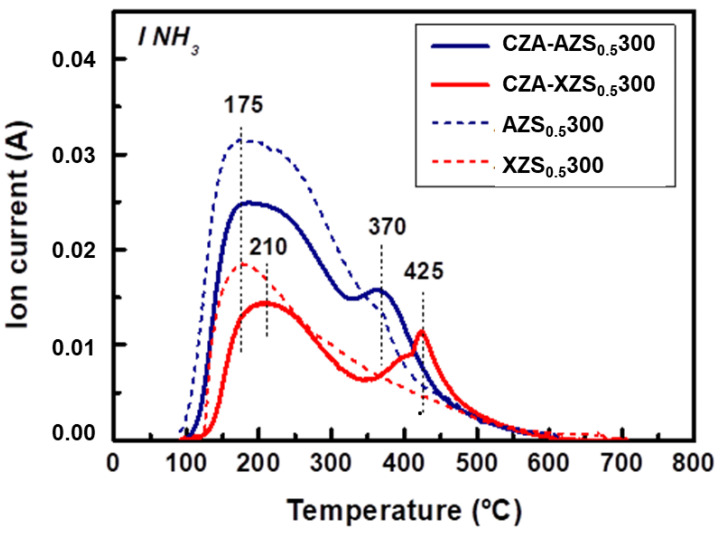
NH_3_-TPD/MS profiles (m/e = 17) of aerogel (CZA-AZS_0.5_300) and xerogel (CZS-XZS_0.5_300) bifunctional composite catalysts. For comparison purposes, the NH_3_ desorption profiles of the corresponding acid catalysts are included.

The acidity changes of the sulfated zirconia after physical mixing with the CZA catalyst were studied using TPD/MS of adsorbed ammonia. Figure 9 shows the ammonia desorption profiles of the CZA-AZS_0.5_300 and CZA-XZS_0.5_300 bifunctional catalysts compared with their corresponding acid catalysts calcined at 300 °C. Based on the ammonia desorption temperature, the amount of acid sites of all samples follows the following trend: weak acid sites (<200 °C) > medium strength acid sites (200 < T < 400 °C) >> strong strength acid sites (>400 °C). By applying the Gaussian deconvolution technique to the profiles, the amount of acid sites was quantified, considering the peak’s areas and the sample weight (Table 3). As compared with the pure aerogel and xerogel sulfated zirconia, both composite catalysts exhibit lower acidity. The decrease in acidity with respect to the pure sulfated zirconia could be explained by the blocking of the acid sites by the CZA particles, thus restricting the access of ammonia to the acid sites. However, both the CZA-AZS_0.5_300 and CZA-XZS_0.5_300 composite catalysts maintained low and moderate acidity (Table 3). Despite the loss of acidity, the CZA-aerogel composite showed a higher acidity than its CZA-xerogel counterpart (Table 3).

**Figure 10:**
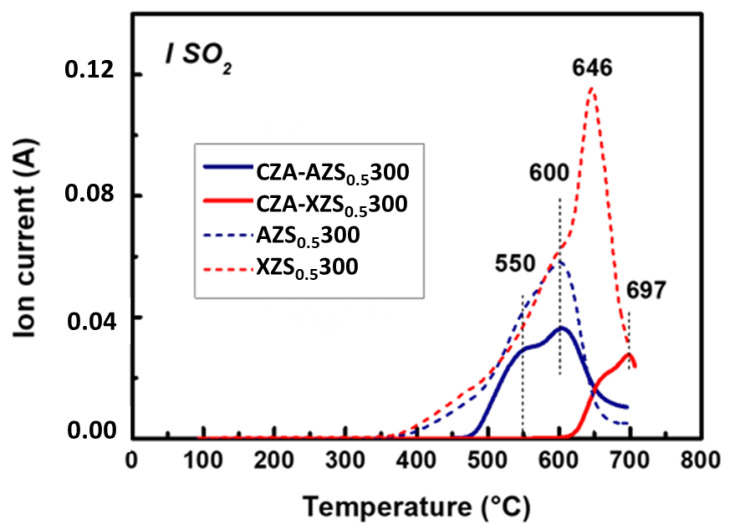
SO_2_ signals (m/e = 64) recorded during NH_3_-TPD experiments of aerogel (CZA-AZS_0.5_300) and xerogel (CZS-XZS_0.5_300) bifunctional composite catalysts. For comparison purposes, the SO_2_ signals of the corresponding acid catalysts are included.

The physical mixing of the CZA catalyst with the aerogel or xerogel sulfated zirconia also changes the thermal behavior of the surface SO_4_^2−^ groups, as shown in Figure 10. The temperature of the evolution of the sulfate groups was more similar for the CZA-AZS_0.5_300 bifunctional composite catalyst than for the pure aerogel sulfated zirconia, indicating that these groups are not strongly modified after their combination with the CZA catalyst. It is noteworthy that, compared to the CZA-aerogel composite, the CZA-xerogel counterpart show the decomposition of the sulfate groups at higher temperatures, which is probably related to their location within the internal structure of the xerogel and/or to a stronger interaction with Zr^4+^ ions after its combination with the CZA catalyst.

#### 3.2.4. Temperature-Programmed Reduction (TPR-H_2_) 

**Figure 11:**
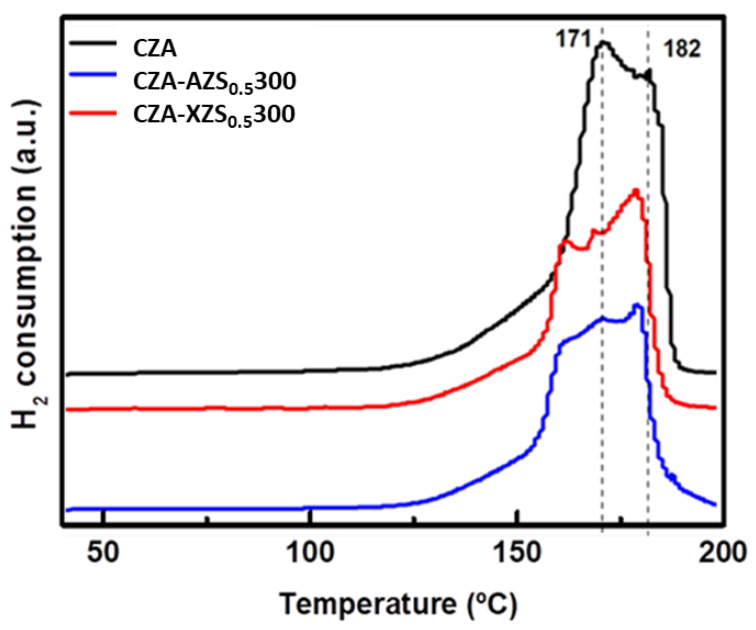
H_2_-TPR profiles of aerogel (CZA-AZS_0.5_300) and xerogel (CZS-XZS_0.5_300) bifunctional composite catalysts. CZA catalyst is included as reference.

The reducibility of the CuO and ZnO species of the aerogel (CZA-AZS_0.5_300) and xerogel (CZA-XZS_0.5_300) bifunctional composite catalysts were estimated using a temperature-programmed reduction with H_2_. Figure 11 represents the hydrogen consumption profile of both composite catalysts and the CZA catalyst used as a reference.

All the reduction profiles presented two overlapping peaks below 200 °C. For the CZA catalyst, the first reduction peak was detected due to the reduction of dispersed CuO in the residual carbonate species that remained after calcination, followed by the second H_2_ consumption peak at around 182 °C, which is typical of the reduction of highly dispersed CuO in close contact with ZnO [25,59]. The shoulder of the main reduction peak also has contributions from the partial reduction of ZnO to ZnO_1−x_. When CZA was mixed with acid catalysts in the bifunctional composite catalysts, the temperature of the reduction peaks shifted to a lower temperature due to the enhanced reducibility of the copper oxide species. The first H_2_ consumption peak in the bifunctional catalysts, corresponding to the residual copper carbonate species, was broadened and decreased with respect to the CZA reference, which could be attributed to the migration of the water on the surface of the sulfated zirconia to CZA, causing the rehydration of the copper carbonate species and altering its reducibility [60]. Compared to the bifunctional catalysts, the pure CZA catalyst displayed the highest hydrogen consumption, showing a good correlation with a higher copper surface area (Table 4). In the studied TPR temperature range, the sulfated species cannot be decomposed below 300 °C, as shown in the NH_3_-TPD results.

#### 3.2.5. Structure of Reduced CZA-ZrO_2_/SO_4_ Bifunctional Composite Catalysts

**Figure 12:**
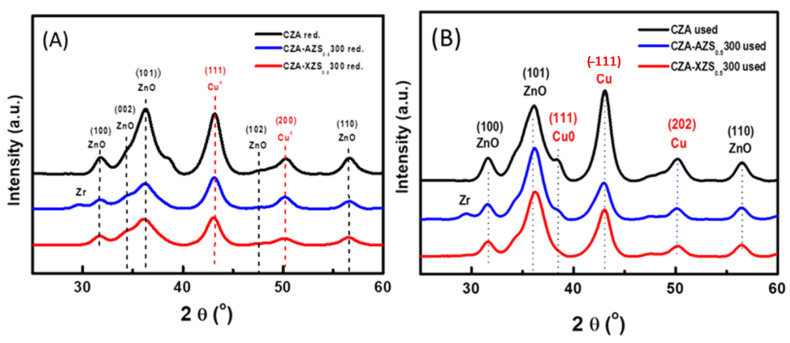
XRD patterns of H_2_ reduced (**A**) and used (**B**) aerogel (CZA-AZS_0.5_300) and xerogel (CZS-XZS_0.5_300) bifunctional composite catalysts. CZA catalyst is included as reference.

The XRD patterns of the reduced CZA reference and the bifunctional composite catalysts are presented in Figure 12A. The reduced CZA catalyst reference showed diffraction peaks related to metallic copper (JCPDS 001-1241) at 2*θ* = 43.15° and 50.27°, and diffraction peaks related to the ZnO hexagonal phase at 2*θ* = 31.75°, 34.38°, 36.24°, and 56.58° (JCPDS 36-1451). For all the samples, the XRD profiles only showed the peaks related to metallic copper, indicating the complete reduction of CuO. The diffractograms of the reduced CZA-AZS_0.5_300 and CZA-XZS_0.5_300 bifunctional composite catalysts showed a lower intensity of the Cu and ZnO diffraction peaks than the CZA reference due to the dilution with the acid sulfated zirconia. As in the case of the calcined samples, small diffraction peaks associated with the tetragonal zirconia phase at 2θ = 30° was observed in the diffraction profile of the CZA-AZS_0.5_300 reduced sample, which means that the reduction process does not produce changes in their crystallization

The calculation of the crystallite size of the copper using the Debye–Scherrer equation, using the Cu (111) diffraction peak at 2*θ*= 43.15°, shows that after reducing treatment under H_2_ at 200 °C, the crystallite domains of Cu slightly increase in all catalysts (Table 4) (from 4.8–5.4 nm to 6.2–6.6 nm), with respect to the size of CuO in the calcined form. It is noteworthy that the Cu particle size in the CZA and bifunctional catalysts is maintained after reduction, which may be due to the careful heat treatment used during the reduction and the good CuO and ZnO nanostructures in the calcined CZA catalyst, which may lead to the growth of Cu particles. The crystallite domains of metallic copper in the reduced CZA-AZS_0.5_300 and CZS-XZS_0.5_300 bifunctional catalysts do not change with respect to the CZA reference. The absence of changes in the crystalline domains of Cu^0^ and ZnO in the bifunctional catalysts does not affect their contacts and the Cu-ZnO synergy responsible for the activity in the methanol synthesis. As shown in Table 4, the crystallite size of the reduced ZnO increases slightly in all catalysts with respect to the size of ZnO in the calcined form. For comparison with the XRD data, the Cu particle size of the freshly reduced catalysts was calculated using the N_2_O chemisorption data (Table 4). As expected, the Cu particle size, determined using the N_2_O chemisorption technique, was slightly larger than that obtained using the XRD, due to the fact that the N_2_O chemisorption technique evaluates both Cu^0^ and the partially reduced ZnO_x_ in close contact with Cu.

The evolution of the Cu-ZnO contacts in the reduced bifunctional composite catalysts CZA-AZS_0.5_300 and CZS-XZS_0.5_300 with respect to the CZA reference was evaluated using the N_2_O chemisorption technique, which selectively titrates the metallic copper atoms in contact with the reduced Zn species. The values of the surface area of the copper, determined using the N_2_O chemisorption technique, are listed in Table 4. As can be seen, the CZA reference has a high specific Cu surface area (45.1 m^2^/g), which indicates a good dispersion between Cu and ZnO, and is consistent with the values that are usually reported for high activity methanol synthesis catalysts. The specific Cu surface area was similar for the reduced bifunctional composite catalysts CZA-AZS_0.5_300 (33.5 m^2^/g) and CZS-XZS_0.5_300 (36.9 m^2^/g) and decreased (around 25%) compared to the CZA reference. The observed decrease in the specific Cu surface area after the physical mixing with the sulfated zirconia was similar for both bifunctional composite catalysts. This decrease in the Cu surface area in the bifunctional catalysts is surprising considering that their XRD data showed no significant variations in the size of Cu^0^ and ZnO with respect to the reference CZA catalyst (Figure 12A and Table 4). The decrease could be probably attributed to a physical blockage of Cu sites by the sulfated zirconia. The reason for this is that the modifications of the Cu-ZnO contacts associated with the Cu^2+^/Zn^2+^ exchange and the acid sites of the sulfated zirconia could be disregarded, as they were minimal according to the H_2_-TPR (Figure 11) and XRD results (Figure 12A).

### 3.3. Catalytic Activity in the Direct Synthesis of DME from Syngas

**Figure 13:**
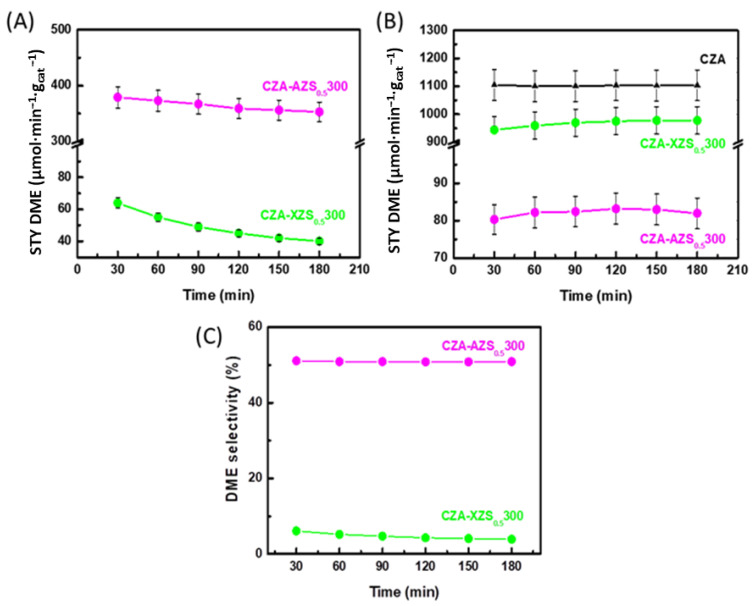
DME (**A**), methanol (**B**) space-time yield (STY) and (**C**) DME selectivity in direct DME formation from syngas over aerogel (CZA-AZS_0.5_300) and xerogel (CZS-XZS_0.5_300) bifunctional composite catalysts. Reaction conditions: T = 250 °C, P = 30 bar).

The bifunctional composite catalysts CZA-AZS_0.5_300 and CZA-XZS_0.5_300 have been evaluated in the direct synthesis of dimethyl ether (DME) from syngas (4.5% CO_2_, 22% CO, 58.8% H_2_ and 14.7% N_2_). The measurements of the catalysts were performed in a fixed bed flow reactor at 250 °C and 30 bar total pressure. Prior to the reaction, both catalysts were activated by a reduction in H_2_/Ar at 200 °C for 2 h. Figure 13A and 13B show the time evolution of the DME and methanol production, expressed as space-time yield (STY), over CZA-AZS_0.5_300 and CZA-XZS_0.5_300 catalysts, respectively. Table 5 summarizes the averaged values of the CO conversion, selectivity (to DME, methanol and CO_2_), and productivity (to DME and methanol) over the CZA reference and bifunctional composite catalysts after 180 min time-on-stream. DME, methanol, CO_2_, and water were the only products detected in the direct synthesis of DME, with no hydrocarbon formation.

Therefore, mixing the CZA catalyst with the acid solids directly produces DME by the common mechanism, suggesting that the following reactions would occur: (8) the conversion of synthesis gas to methanol, followed by (9) the dehydration of the latter to yield dimethyl ether (DME), and (10) the water–gas shift reaction, as shown in [61]:

2 CO + 4H_2_ ↔ 2CH_3_OH       ∆H = −181 kJ (8)

2 CH_3_OH ↔ CH_3_OCH_3_ +H_2_O       ∆H = −23 kJ (9)

H_2_O + CO ↔ H_2_ +CO_2_       ∆H = −41 kJ (10)

As expected, the metallic CZA catalyst demonstrated an exceptional methanol yield (1102 µmol_CH3OH_·min^−1^·g_cat_^−1^) and 100% of the methanol selectivity, which is consistent with the highest reported data in the literature. As can be seen, the mesoporous aerogel-containing composite was more effective for the DME production than its xerogel-containing counterpart. In fact, the CZA-AZS_0.5_300 catalyst exhibits a DME selectivity of 50.9% compared to the DME selectivity of 9.4% observed in the CZA-XZS_0.5_300 catalyst (Figure 13C) at carbon monoxide conversions of 29.8 and 26.8%, respectively. After first 60 min of the catalyst stabilization, the DME production in the CZA-AZS_0.5_300 and CZA-XZS_0.5_300 catalysts decreased slowly, suggesting a catalyst deactivation by the produced water. The decrease of the DME production for the CZA-AZS_0.5_300 catalyst was 11.6 µmol/h, whereas for the CZA-XZS_0.5_300 catalyst, the DME production was lower, at 3 µmol/h. Nevertheless, it is noticeable that the DME yield slightly decreases in the case of the aerogel-containing catalyst, but without any change in the DME selectivity during the reaction time (Figure 13C). This indicates the stability of the acid sites of the CZA-AZS_0.5_300 catalyst under reaction conditions.

To have a measure of the results obtained, the DME yield of the most active bifunctional composite catalyst (CZA-AZS_0.5_300) is compared in Table 6 with some of the most active bifunctional catalysts reported in the bibliography. As shown in this table, our CZA-AZS_0.5_300 bifunctional composite catalyst shows a twofold higher DME production from syngas compared to other composite catalysts based on zeolites [62] and heteropolyacid catalysts [25].

To date, the Cu/ZnO/Al_2_O_3_–HZSM-5 hybrid catalytic system is considered the best formulation for the direct synthesis of DME. Unfortunately, this catalyst suffers from deactivation with time-on-stream due to water-induced copper sintering, Cu ion exchange with zeolite, or coke deposition [55,63,64].

## 4. Discussion

The overall characterization of the CZA-ZrO_4_/SO_4_^2−^ bifunctional composite catalysts showed that the main difference between the aerogel and xerogel composite catalysts lies in the texture, acidity, and stability of the SO_4_^2−^ groups of the sulfated zirconia. These differences were due to the different preparation methods of the sulfated zirconia: the aerogel was obtained using gel drying under supercritical conditions, whereas the xerogel was prepared using thermal drying in a conventional oven. After calcination at an optimized temperature (300 °C), the aerogel presents a mesoporous structure, whereas the xerogel, with its microporous structure, has inferior textural properties (Table 1). The mesoporous structure of the aerogel proved to be an important factor influencing the DME production, with the CZA-aerogel composite being much more active than the CZA-xerogel catalyst. Moreover, based on the BET and XRD results of the pure acid catalysts, it was found that the aerogel sulfated zirconia catalyst possessed a mesoporous structure with a higher specific surface area than the xerogel catalyst, with a small crystallite size and the presence of the tetragonal phase of zirconia, which is considered as an active phase [65,66,67], and which generates more acidity; these factors could also be identified as responsible parameters for the methanol dehydration results obtained. Based on the observation of zeolites (ZSM-5 nanocrystals) as catalysts for methanol dehydration, it has been reported that reducing the crystal size and the developed external surface area both increases the accessibility of the acid sites and the diffusion rate of reactants and products, resulting in stable activity and higher conversions [65,66]. However, the catalytic performance of the composite catalysts is highly dependent on the number of acid sites [11], their strength [11], and the metallic copper surface area [64]. In this scenario, the xerogel catalyst was found to be a poor methanol dehydrogenation catalyst (Table 5), which can be explained by the difficulty of the DME formation in the internal microporous structure of the xerogel. In the case of the CZA-aerogel ZrO_4_/SO_4_^2^ bifunctional composite catalyst, the enhanced DME formation is most likely due to the reduced diffusion path and easier access of methanol to the acid sites of the sulfated zirconia as a result of its mesoporous structure. This result leads to the conclusion that the synergistic effect between the active methanol synthesis sites of the CZA catalyst and the acid sites of the sulfated zirconia is enhanced when the sulfated zirconia has a mesoporous structure. The observed superiority of the mesoporous aerogel-containing composite over its xerogel-containing counterpart is in good agreement with that observed for the hybrid catalyst prepared by milling the Cu/ZnO/Al_2_O_3_ catalyst with HZSM-5 zeolite [67]. In those catalysts, the loss of Brønsted acid sites was explained as due to the blocking of zeolite micropores by metal particles of the catalyst and by the partial exchange of zeolite protons by Zn^2+^ and Cu^2+^ cations of the methanol catalyst [67].

The lower DME selectivity of the CZA-xerogel ZrO_4_/SO_4_^2^ bifunctional catalyst (Figure 13C) can also be explained by its lower concentration of acid sites, as confirmed by the NH_3_-TPD/MS profiles (Table 3). In fact, the combination of the more acidic aerogel sulfated zirconia with the CZA catalyst significantly increases the DME formation (Figure 13A) and decreases the methanol formation rate (Figure 13B). The same acidity–DME selectivity correlation was observed by Ramos et al. for some acid dehydration catalysts, such as alumina, HZSM-5, tungsten–zirconia, and sulfated zirconia [12]; in addition, similar behavior, previously observed in DME production over mixed Cu/ZnO/Al_2_O_3_ and ZSM-5 catalysts, was related to the low concentration of Brønsted acid sites [54].

In this study, both CZA-ZrO_4_/SO_4_^2−^ bifunctional composite catalysts have almost the same copper surface area and the same crystallite size of metallic copper, but different acidic properties, which seems to be the most important factor to determine the activity and selectivity to DME. In this regard, ammonia adsorption followed by a TPD/MS analysis indicated that aerogel sulfated zirconia had a higher acidity than the corresponding xerogel (Table 2). In contrast, in the study by Rachmat et al., it was observed that amorphous sulfated zirconia calcined at 400 °C adsorbs more ammonia than crystalline sulfated zirconia, which was explained by the presence of hydroxyl groups bound to Zr, which are incompletely eliminated during calcination at 400 °C [33]. Since hydroxyl groups are suitable for the chemisorption of ammonia [33], our NH_3_-TPD/MS results suggest that the bifunctional composite catalyst with more crystalline aerogel sulfated zirconia might have a higher amount of hydroxyl groups than its bifunctional composite obtained by the mixing of CZA with an amorphous xerogel. In this regard, it should be emphasized that, unlike xerogel, crystalline aerogel calcined at 300 °C has the strong acid sites required for DME production from syngas. In this respect, this study emphasized the importance of weak and medium acid sites in the composite catalysts for their effectivity in DME synthesis.

Several reaction mechanisms have been presented for methanol dehydration on acid catalysts [12]. The Lewis acidity-based mechanism requires a pair of adjacent acid-based sites for the reaction to take place between the alcohol molecule adsorbed on an acid site and an alkoxide anion adsorbed on a basic site [12]. However, the general belief is that Brønsted sites, which are formed by the addition of sulfate groups to zirconia [68], are responsible for acid-catalyzed reactions being more active and for catalysts having stronger acid sites. In good agreement with the work of Saravanan et al. [69], our aerogel, prepared using the same method as Saravanan et al., has a larger amount of more accessible acid sites than the xerogel due to its higher specific surface area compared to the xerogel (Table 1). Thus, we conclude that the higher DME production from CO_2_ hydrogenation over the aerogel-containing CZA-ZrO_2_/SO_4_^2−^ composite catalyst is due to its higher Brønsted acidity with respect to the xerogel-containing counterpart.

Finally, the decrease in DME production with a simultaneous increase in methanol production observed for the CZA-ZrO_4_/SO_4_^2−^ bifunctional catalyst suggests the deactivation of its acid sites by the generated H_2_O. This loss of yield may be related to detrimental interactions between CZA and the excess water from DME production, as the kinetics of methanol formation are very sensitive to water content. In addition to the inhibitory effect of water on the methanol synthesis kinetics, water can also promote the sintering of copper particles. However, the comparison of the average copper particle size in the catalysts that were used (determined using XRD, Figure 12B) showed that water from a higher DME production does not significantly affect the particle size, as the Cu growth was similar in all the catalysts that were used. The catalyst activity results show that the deactivation in the DME production was higher for the CZA-aerogel ZrO_4_/SO_4_^2^ catalyst than for the CZA-xerogel-ZrO_4_/SO_4_^2^ catalyst. In this regard, the TGA characterization indicates that aerogel sulfated zirconia has a lower hydrophobicity than xerogel sulfated zirconia (Figure 3). Therefore, the higher deactivation observed for the CZA-AZS_0.5_300 sample compared to the CZA-XZS_0.5_300 sample can be attributed to its lower hydrophobicity. However, the deactivation of the catalyst by the differential migration of the active Cu^2+^/Zn^2+^ species to the acid sulfated zirconia by the cationic exchange on the acid sites cannot be excluded [21,67]. In this regard, the deactivation of both the metallic and the acid functions of the catalyst due to the Cu sintering and migration into the zeolite pores was reported by Ordomsky et al. [70]. The higher surface area and porosity of the erogel sulfated zirconia compared to the xerogel counterpart could facilitate the possible migration of Cu^2+^/Zn^2+^ species to the acid sites. This aspect should be taken into account to justify the higher deactivation observed in the case of the bifunctional catalyst with aerogel sulfated zirconia, if this mechanism operates in the observed deactivation. Furthermore, it is noteworthy that in spite the deactivation for the DME production, the selectivity to DME was maintained, which suggests the maintenance of the desired amount of acid sites while potentially deactivating strong acid sites due to the water adsorbing molecules that seem more attracted to Lewis acid sites (Zr^4+^) [14]. The deactivation of the catalyst is currently under study.

Summarizing the results presented, we have shown that the behavior of the bifunctional catalysts prepared by the physical mixing of CZA and sulfated zirconia depends on the mesoporosity of the sulfated zirconia, which causes an improved accessibility of methanol molecules to the acid sites. It is noteworthy that the DME production rate achieved on the bifunctional catalysts prepared with mesoporous sulfated zirconia (STY DME of 356 μmol_DME_·min^−1^·g_cat_^−1^) is among the highest reported in the literature, illustrating the interest of combining both functionalities for the preparation of efficient catalysts for the direct synthesis of DME. The overall results provided valuable insights into the factors influencing DME production and highlighted the importance of the mesoporosity of the acid sulfated zirconia in achieving the optimal efficiency for the direct synthesis of DME on bifunctional composite catalysts based on the combination of CZA and sulfated zirconia catalysts.

## 5. Conclusions

This work presents the application of bifunctional catalysts prepared via the physical mixing of a Cu-ZnO(Al) methanol synthesis catalyst with sulfated zirconia (acidic catalyst) for the direct production of dimethyl ether (DME) from syngas. The enhanced DME production on the CZA-aerogel ZrO_2_/SO_4_ bifunctional composite catalyst was attributed to the cooperative effect of the methanol synthesis and the methanol dehydration acid sites on the sulfated zirconia with optimized textural properties, indicating the importance of the mesoporosity of the acid for the direct DME formation. The main conclusions of this work can be summarized as follows: (i) The influence of the drying method on the textural properties and acidity of the sulfated zirconia, and the hypercritical drying method of sulfated zirconia to obtain aerogels, followed by calcination at 300 °C, is more favorable for DME production than solvent evaporation via thermal heating to obtain xerogels. The latter method negatively affects the acidity and morphology of the sulfated zirconia, which implies a decrease in DME production. (ii) The strength and type of acid sites present in the acid catalyst, together with its well-developed mesoporous and crystalline structures, play crucial roles in the success of the direct synthesis of DME. Also, the proper dispersion of copper in the composite catalyst is important to promote and achieve a high conversion yield of carbon monoxide (CO) to DME. The high activity (STY DME of 356 μmol_DME_·min^−1^·g_cat_^−1^) and stability evidence the effectiveness of this catalytic system for the production of DME from syngas.

## Figures and Tables

**Table 1:** Textural properties ^a^ and TGA ^b^ data of the aerogel and xerogel sulfated zirconia.

Materials	N_2_ Adsorption–Desorption Isotherms ^a^					Weight Loss ^b^ (%)	
	S_BET_ ^c^ (m^2^·g^−1^)	V_total_ ^d^ (m^3^·g^−1^)	V_micro_ ^e^ (m^3^·g^−1^)	S_micre_ ^c^ (m^2^·g^−1^)	*d*_pore_ (nm)	Total	SO_x_
AZS_0.5_	201	0.601	0.001	4.4	12.0	58.7	28.6
AZS_0.5_300	126	0.372	0.005	10.7	11.9	34.3	10.7
AZS_0.5_560	103	0.355	0.003	8.2	13.8	22.4	12.8
XZS_0.5_	169	0.203	0.023	46.3	4.8	19.4	12.2
XZS_0.5_300	116	0.136	0.017	33.1	4.7	25.8	11.3
XZS_0.5_560	52	0.083	0.002	4.7	6.4	50.8	17.8

^c^ BET method; ^d^ volume adsorbed at P/P_0_ = 0.99; ^e^ t-plot.

**Table 2:** Quantitative data from NH_3_-TPD/MS profiles of aerogel and xerogel sulfated zirconia.

Catalysts	Total Acidity	Amount of Acid Sites (area/g_cat_)		
		Weak 200 < T	Moderate 250 < T < 400	Strong >400 °C
AZS_0.5_	7.4	2.2	5.1	0.1
AZS_0.5_300	6.8	1.1	5.6	0.1
AZS_0.5_560	5.1	0.7	1.8	2.6
XZS_0.5_	4.2	1.1	2.6	0.5
XZS_0.5_300	3.5	1.1	1.8	0.6
XZS_0.5_560	0.9	0.3	0.3	0.3

**Table 3:** Quantitative data from NH_3_-TPD/MS profiles of CZA-AZS_0.5_300 and CZA-XZS_0.5_300 bifunctional composite catalysts.

Catalysts	Total Acidity	Amount of Acid Sites (area/g_cat_)		
		Weak 200 < T	Moderate 250 < T < 400	Strong >400 °C
CZA-AZS_0.5_300	5.8	0.9	4.8	0.1
AZS_0.5_300	6.8	1.1	5.6	0.1
CZA-XZS_0.5_300	3.5	0.6	1.6	1.3
XZS_0.5_300	3.5	1.1	1.8	0.6

**Table 4:** Crystallites size (from XRD) and specific surface of copper (from N_2_O chemisorption) for the calcined, reduced and used CZA and bifunctional composite catalysts.

		Crystallite Size (nm)		Cu^0^ Surface Area (m^2^/g_cat_)
		CuO(Cu)	ZnO	
CZA	calcined	5.4	6.0	45.1
	reduced	6.3 (6.5) ^a^	8.0	
	used	7.4	8.2	
CZA-AZS_0.5_300	calcined	4.9	5.4	33.5
	reduced	6.2 (8.7) ^a^	6.7	
	used	6.4	8.1	
CZA-XZS_0.5_300	calcined	4.8	4.2	36.9
	reduced	6.6 (7.9) ^a^	8.8	
	used	6.6	5.7	

^a^ crystallite size calculated considering N_2_O chemisorption data.

**Table 5:** Averaged values of CO conversion, selectivity (to DME, methanol and CO_2_), and productivity (STY to DME and methanol) over the CZA reference and aerogel (CZA-AZS_0.5_300) and xerogel (CZS-XZS_0.5_300) bifunctional composite catalysts.

Catalyst	CO Conversion (%)	Selectivity (%)			STY DME	STY CH_3_OH
		CH_3_OH	DME	CO_2_		
CZA	20.9	100	-	-	-	1102
CZA-AZS_0.5_300	29.8	8.3	50.9	40.9	364	82
CZA-XZS_0.5_300	26.8	82.5	9.4	8.1	52	980.4

**Table 6:** Comparison of the direct DME production from syngas of the most active bifunctional catalyst prepared (CZA-AZS_0.5_300) with some of the most active bifunctional catalysts previously reported in the bibliography and measured under similar reaction conditions.

Catalyst	T (°C)/P (bar)	CO Conv. (%)	Selectivity (%)			Yield DME (%)	Ref.
			CH_3_OH	DME	CO_2_		
CZA-AZS_0.5_300	250/30 ^a^	29.8	8.3	50.9	40.9	15.2	This work
CZA-HZSM-5	250/30 ^a^	11.4	6.2	55.7	38.1	6.3	[25]
CZA-2.7HPW/Ti	250/30 ^a^	11.0	6.4	53	40.6	5.8	[25]
CZA-FER	250/40 ^b^	30.2	42.8	28.7	27.8	8.7	[62]
CZA-NaY	250/40 ^b^	14.6	71.7	12.5	15.2	1.8	[62]
CZA-HY	250/40 ^b^	22.7	12.5	29.7	57.2	6.7	[62]
CZA-ZSM-5	250/40 ^b^	13.9	64.1	14.4	20.9	2.0	[62]
CZA-FER	250/40 ^b^	30.2	42.8	28.7	27.8	8.7	[62]

^a^ CO/CO_2_/H_2_ = 22/4.5/58.8 mol.%; ^b^ CO/CO_2_/H_2_ = 41/21/38 vol.%.

## Data Availability

Data are contained within the article.
